# Impact of COVID-19 Pandemic on Hand Surgery Volume in Japan

**DOI:** 10.3390/jcm14051518

**Published:** 2025-02-24

**Authors:** Hidemasa Yoneda, James Curley, Katsuyuki Iwatsuki, Masaomi Saeki, Nobunori Takahashi, Michiro Yamamoto

**Affiliations:** 1 Department of Human Enhancement and Hand Surgery, Nagoya University Graduate School of Medicine, 65 Tsurumai-cho, Showa-ku, Nagoya 466-8550, Japan; 2 Department of Orthopedic Surgery, Aichi Medical University, 1-1 Yazakokarimata, Nagakute 480-1195, Japan

**Keywords:** COVID-19, upper extremity, hand surgery, surgical volume, hospitalization, surgical procedures

## Abstract

**Objectives:** The impact of the COVID-19 pandemic on hand surgery in Japan has not been fully elucidated. This study investigated changes in the volume of hand surgery practiced during the pandemic. **Methods:** We used the National Database Open Data Japan (NDB-ODJ), a comprehensive repository of healthcare data administered by the government, to investigate changes in the volume of hand surgery services delivered during the COVID-19 pandemic. The type and number of upper extremity surgical procedures was examined during each month of the pandemic to identify associations. **Results:** During the first wave in the spring of 2020, scheduled surgeries decreased by 44% compared to pre-pandemic levels, with arthroplasties, osteotomies, and polydactyly surgeries experiencing the largest reductions. Trauma surgeries remained relatively stable, and some procedures like tendon repair and replantation even increased. While overall surgical volumes recovered in the second half of the pandemic, certain procedures, including finger pinning and tendon repair, remained below pre-pandemic levels. Interestingly, surgeries for Dupuytren contracture and amputation increased compared with the pre-pandemic period. Many scheduled and emergency procedures shifted to outpatient surgeries during the pandemic, and the proportion of inpatient surgeries decreased. In particular, the proportion of outpatient surgeries increased significantly in open reduction and internal fixation for wrist and forearm fractures, as well as in amputation surgeries. **Conclusions:** The COVID-19 pandemic had a minimal impact on the volume of hand surgery conducted in Japan, with a decrease in elective surgeries only during the first wave in the spring of 2020. Notably, the pandemic triggered a shift from inpatient to outpatient surgery for many procedures.

## 1. Introduction

The COVID-19 pandemic, which emerged in 2020, had a significant impact on surgical treatments worldwide. Lockdowns were implemented in many countries to curb the spread of the virus, imposing substantial restrictions on people’s social lives. The reduction in group activities and outdoor pursuits led to a marked decrease in sports-related injuries and, consequently, a significant decline in the number of patients seeking treatment for traumatic injuries [1,2].

Healthcare resources were reallocated to prioritize the treatment of critically ill patients with respiratory failure. Medical supplies were preferentially used for the prevention and treatment of COVID-19, and masks and ethanol became temporarily scarce. Some general wards were converted into infectious disease wards, and intensive care physicians and pulmonologists were dedicated to treating the surge of COVID-19 patients. To compensate for the burden of long, challenging shifts, patient surges, and personnel shortages, in part due to their own infections, cross-departmental staffing was implemented [3].

Following the declaration by the World Health Organization (WHO) of a Public Health Emergency of International Concern on 30 January 2020, the American College of Surgeons recommended postponing elective surgeries except for emergency procedures on 13 March 2020. Based on these pronouncements, practitioners of orthopedic surgery in Japan, primarily led by the Japanese Orthopedic Association, also responded. Surgeons swiftly adapted to the pandemic situation by adjusting hospital resources and postponing elective surgeries [4].

In the United States, the number of hand surgeries significantly decreased, particularly during the emergence of challenging COVID-19 variants and surges in cases [5,6]. In the United Kingdom, there was an increase in the proportion of non-operative management and remote follow-up for hand trauma patients [7]. In India, there was also an increase in the proportion of conservative treatment for the management of hand trauma [8].

Studies show that Japan successfully contained COVID-19 early on without implementing lockdowns or mandatory restrictions on behavior [9,10]. Notwithstanding this, reports indicate a general decline in surgical volumes in Japan during the pandemic [11,12]. To our knowledge, the impact of the COVID-19 pandemic specifically on hand surgery treatment in Japan has not been reported.

Hand surgery specialists are responsible for treating patients suffering from acute trauma, a substantial responsibility given that the number of those requiring intervention may be comparable to, or even greater than, those seeking elective surgeries. Irrespective of other factors, the volume of trauma patients presenting to a healthcare system follows predictable, generally stable trends. Postponing or redirecting such regularly occurring urgent care to allocate medical resources to critically ill COVID-19 patients posed particular challenges.

We became interested in how the pandemic affected the overall practice of hand surgery, including trauma and elective procedures, and wanted to document how healthcare professionals navigated these challenges. Therefore, we aimed to investigate and elucidate the effects of the COVID-19 pandemic on hand surgery in Japan.

## 2. Materials and Methods

### 2.1. Data Source

We utilized data sets from the National Database of Health Insurance Claims and Specific Health Checkups of Japan (NDB) [13]. Japan has universal health coverage and claims are reimbursed through its statutory health insurance system. Anonymized, aggregated data from the NDB, published annually as NDB Open Data Japan (NDB-ODJ), include statistics on medical procedures and pharmaceuticals dispensed and can be downloaded from the website of the Ministry of Health, Labour, and Welfare of Japan (MHLW). Data up to March 2023 are currently available. The NDB-ODJ is a comprehensive database covering all hospitals with 20 beds or more; however, it does not include statistics on claims arising from occupational and motor vehicle injuries. Therefore, we do not have a complete understanding of the treatment trends for patients utilizing these insurance schemes.

### 2.2. Definition of the Pandemic Period

The novel coronavirus was first reported in Wuhan, China, in December 2019. As previously mentioned, the WHO declared a Public Health Emergency of International Concern in January 2020 and characterized it as a pandemic that March. The present study tracks with the pandemic periods determined by the MHLW (Table 1) in which successive waves of the pandemic were determined based on the number of patients who were receiving intensive care for COVID-19 [14]. While acknowledging that the pandemic was not over, the WHO withdrew its emergency declaration in May 2023, legally mandated infection control measures were lifted, and COVID-19 began to be managed more akin to seasonal influenza. Therefore, for this study, we elected to define this date as the end of the pandemic. The pandemic periods detailed in Table 1 were analyzed in two distinct phases. The initial phase encompasses periods 1 through 5, while the later phase covers periods 6 through 8.

### 2.3. Analytic Methods and Statistical Processing

We extracted the total number of upper extremity surgical procedures as a representation of hand surgery performed. The specific procedures are listed in Table 2. These were broadly classified into elective surgeries and urgent surgeries performed for trauma, vascular compromise, and other acute conditions. The number of surgical procedures was investigated on a monthly basis. The number of new COVID-19 cases and the number of critically ill patients were extracted from the MHLW website [15].

In the NDB-ODJ, counts of 10 or fewer are not reported. Therefore, we forcibly entered a value of 5 in such cases. Based on the values, we investigated the following:

- Changes in the total number of surgical procedures during the pandemic.
- Comparison of the number of surgical procedures before and after the first pandemic period.
- Comparison of the number of surgical procedures before the pandemic, in the first half (initial phase) of the pandemic, and in the second half (later phase) of the pandemic.
- Changes in the number of outpatient surgical procedures before the pandemic, in the first half (initial phase) of the pandemic, and in the second half (later phase) of the pandemic.

For statistical processing, the Mann–Whitney *U* test was used for comparisons between two groups. Spearman’s rank correlation coefficient was used to analyze the correlation between two variables. The Kruskal–Wallis test was used to assess the distribution among three or more groups, while Dunn’s test with Bonferroni adjustment was performed as a post-hoc test. In all tests other than those with a Bonferroni adjustment, a *p*-value of less than 0.05 was considered statistically significant. IBM SPSS Statistics 29.0 (Armonk, NY, USA) was used for the statistical analysis.

The items analyzed included: trends in the number of critically ill COVID-19 patients, new infections, and surgical procedures; changes in surgical procedures during the first period of the pandemic; the number of surgical procedures before and after the pandemic; and the proportion of outpatient and inpatient treatments before and after the pandemic.

## 3. Results

### 3.1. Trends in the Number of Critically Ill Patients, New Infections, and Surgical Procedures

**Figure 1:**
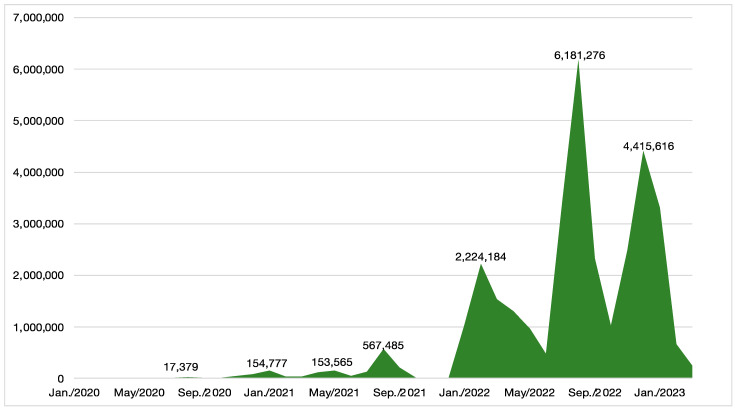
Trends in the number of new infections. The precise number of new infections is shown at each peak.

**Figure 2:**
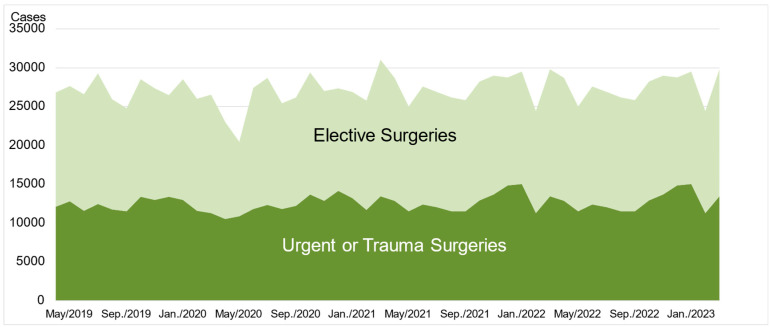
Trends in the numbers of all surgical procedures.

Figure 1 shows the trends in the number of critically ill patients and new infections. Figure 2 shows the trends in the numbers of all surgical procedures investigated in this study. The number of surgical procedures for the examined items decreased by more than 10% in April and May 2020 during the first wave of the pandemic, and in May 2021 and May 2022. These decreases were related to the prevalence of variant COVID-19 strains (Figure 2), but there was no correlation between the number of infected individuals, critically ill patients, and procedures. The largest decrease occurred during the first wave in April and May 2020, with no significant fluctuations observed thereafter.

### 3.2. Change Between Surgical Procedures Before and After the First Pandemic Period

Regarding changes in the number of surgical procedures during the first pandemic period, a comparison between January 2020 (immediately before the pandemic) and May 2020 revealed that the average decrease in the number of elective surgeries was 44%, with nearly half of these procedures being reduced (Table 3). In contrast, the number of trauma surgeries remained relatively stable. Among elective surgeries, the number of arthroplasty, osteotomy, and polydactyly surgeries decreased to less than half. While some trauma and emergency surgeries decreased during this period, the number of tendon suture, amputation, nerve suture, and replantation procedures increased.

### 3.3. Changes in Surgical Volume Before and After the Pandemic

Compared to the pre-pandemic period, the volume of many surgical procedures decreased during the first half of the pandemic. Among elective surgeries, significant decreases were observed in osteotomies, while among trauma and emergency surgeries, there were significant decreases in finger pinning, as well as in open reduction and internal fixations (ORIF) (Table 4). Other surgical procedures showed only minor fluctuations. There were notable differences, however, in the number of Dupuytren contracture and amputation surgeries, which increased compared to the pre-pandemic period. Overall, the rates of many of the surgical procedures that were negatively impacted in the first half of the pandemic recovered to normal in the second half.

### 3.4. Changes in the Proportion of Outpatient Surgeries

There was a shift towards outpatient surgery for many elective and urgent surgical procedures, with a decrease in the proportion of inpatient surgeries (Table 5). Among elective surgeries, the proportion of outpatient procedures increased significantly for tenolysis, arthroplasty, carpal tunnel release, ulnar nerve transposition, and Dupuytren contracture surgery. Among trauma and emergency surgeries, a significant increase was observed in outpatient procedures for open reduction and internal fixation of wrist and forearm fractures as well as amputations. These trends continued into the second half of the pandemic.

## 4. Discussion

In this study, we investigated the impact of COVID-19 on the practice of hand surgery in Japan. We found that the number of trauma surgeries was largely unaffected by the pandemic, although there were some fluctuations. It is highly likely that the demand for surgery in the hand region was met during this period, even amidst reports suggesting that the incidence of trauma decreased. We observed a decrease in the number of non-traumatic cases during the first period of the pandemic, but these numbers recovered in the second period and remained stable thereafter.

Initially, we anticipated that the number of surgeries would have either remained the same or decreased after the onset of the pandemic compared to before, in line with reports from around the world. However, when examined by category, the number of surgeries during the pandemic significantly increased for two procedures: “fingertip amputation” and “Dupuytren contracture surgery”. It is possible that surgeons, impelled to prioritize and reallocate medical resources, resorted to performing fingertip amputation rather than replantation, as the former is simpler and requires a shorter operative time. However, since the number of replantation surgeries did not change significantly during the same period, we presume that the increase was influenced by the rise in finger gangrene cases associated with the increase in COVID-19 patients. We attributed the increase in the number of Dupuytren contracture surgeries to the commercial withdrawal of the Collagenase *Clostridium histolyticum* (CCH) injection just before the pandemic, forcing patients who were eligible for pharmacological treatment to opt for surgery [16].

The pandemic in Japan led to a shift in hand surgery practice, with a transition from inpatient to outpatient care when feasible. In Japan, fees for medical services are set by the government. Patients bear responsibility for contributing a certain percentage of the total cost, but there is monthly cumulative cap on out-of-pocket expenses. Therefore, the typical direct cost paid by a patient for inpatient treatment does not differ significantly from that of outpatient treatment. Moreover, hospitals have a financial incentive to provide inpatient surgery because the reimbursable billing amount is higher for inpatient compared to outpatient treatment. For these reasons, patients with concerns ranging from managing postoperative pain to inconvenience due to short-term follow-up have often been encouraged to choose inpatient surgery, even for conditions that would commonly be treated on an ambulatory basis in other countries. For example, in 2019, just before the pandemic, around 40% of patients undergoing carpal tunnel release were treated on an inpatient basis. However, during the COVID-19 pandemic, the reallocation of medical staff and hospital beds made it necessary to minimize inpatient admissions. Additionally, to avoid the risk of nosocomial infections, there was a shift towards day surgery and outpatient care.

While approximately 80% of hand trauma treatment in the United States is performed under general anesthesia [17], most hand trauma surgeries in Japan are performed under local or regional anesthesia, eliminating the need for recovery room facilities, making the transition to outpatient surgery easier. Although we were unable to investigate it in this study, the duration of hospital stays may also have been affected. In the United States, the length of hospitalization for patients undergoing lower extremity joint arthroplasty was shortened to 1.7 days during the pandemic [18]. The shift to outpatient care and reduction in length of stay for inpatient admissions were natural consequences of maintaining surgical services during the pandemic.

Among the upheavals engendered by COVID-19, in Japan, the pandemic appears to have marked an inflection point for providers, patients, and payers of healthcare services. We believe that the shift from inpatient to outpatient hand surgeries, a direct if unanticipated effect of the pandemic, will be sustained. In April 2024, the Ministry of Health, Labour, and Welfare significantly reduced the “Short term stay surgery basic fee” applied to hospitalizations of several days [19]. With the loss of profits from inpatient services, hospitals will likely encourage patients to opt for outpatient rather than inpatient care. Furthermore, from 2025, the out-of-pocket costs to consumers will be gradually increased and, depending on annual income, are expected to almost double for some patients [20]. We predict that the shift to outpatient surgery will further accelerate as both patients and healthcare providers face increased burdens and reduced benefits from inpatient care.

Will Japanese healthcare professionals be able to utilize the lessons learned when a similar pandemic occurs in the future? Basic infection control measures were thoroughly implemented among healthcare professionals. When severe COVID-19 cases increased, non-urgent treatments were suspended, and medical resources were inevitably triaged to focus on urgent and critically ill patients. As a result, Japan was able to control the COVID-19 pandemic relatively quickly compared to other countries. The shift to outpatient surgery, although an unintended consequence, is a trend that will likely continue, with careful consideration of the necessity of hospitalization and minimizing the length of stay to reduce nosocomial infections and contain costs.

This study has several limitations. As described previously, claims arising from occupational and motor vehicle injuries are not included in the NDB-ODJ, and the study lacks understanding of the treatment trends for patients utilizing these insurance schemes. Additionally, there is a possibility of aggregation errors due to coding inconsistencies inherent in such insurance reimbursement databases. While the NDB-ODJ has the advantage of being unrestricted and generally accessible, the data are released after an approximate two-year lag, making timely reporting challenging. Despite these limitations, this study was able to clarify the impact of the COVID-19 pandemic on hand surgery.

## 5. Conclusions

Overall, the COVID-19 pandemic had a minimal impact on the volume of hand surgery in Japan. By 2023, the number of most types of hand surgery procedures had recovered to pre-pandemic levels. However, there was a notable increase in amputation and Dupuytren contracture surgeries compared to the pre-pandemic period. The number of patients who experienced treatment delays due to the pandemic was low compared to other countries. Additionally, the concentration of medical resources during the pandemic led to a shift towards outpatient treatment for many procedures in hand surgery.

## Figures and Tables

**Table 1:** Periods of the COVID-19 pandemic determined by the MHLW [10] and the corresponding phases used in this study.

Period	Start and End Date	Study Phases
1st	13 January 2020–7 June 2020	Earlier
2nd	8 June 2020–27 September 2020	
3rd	28 September 2020–28 February 2021	
4th	1 March 2021–20 June 2021	
5th	21 June 2021–28 November 2021	
6th	29 November 2021–19 June 2022	Later
7th	20 June 2022–9 October 2022	
8th	10 October 2022–8 May 2023	

MHLW, Ministry of Health, Labour, and Welfare of Japan.

**Table 2:** Surgical procedures analyzed.

Surgical Procedure
Elective
Tenosynovectomy (K028)
Tenolysis (K035)
Tendon transfer (K040)
Capsular release of elbow, wrist, and hand (K076-2)
Arthroplasty of hand and digits (K080)
Rotator cuff repair (K080-3, 4)
Stabilization of shoulder (K080-5)
Osteotomy for wrist and forearm fracture malunion (K054, K057)
Arthroscopic synovectomy of wrist and hand (K066-2)
Carpal tunnel release (K093)
Ulnar nerve transposition for cubital tunnel syndrome (K197)
Fasciectomy of the cord for Dupuytren contracture (K099-2)
Reconstruction of syndactyly or polydactyly (K100, 101)
Urgent or trauma
Tendon suture (K037)
Stabilization of subluxated extensor tendons (K040-2)
Percutaneous pinning for fracture of digits (K045-3)
ORIF for forearm and wrist fractures (K046-2, K073-2)
ORIF for humerus and elbow fractures (K046-1, K073-1)
ORIF for scaphoid fractures (K046-2)
ORIF for fractures of a digit (K046-3)
Finger amputation (K086, K087)
Replantation of finger (K088)
Nerve repair (K182)

Numbers starting with “K” are surgical billing codes used by the Japanese insurance system. ORIF, open reduction and internal fixation.

**Table 3:** Changes in the numbers of surgical procedures between January and May 2020.

Surgical Procedure	Ratio of Change
Elective	
Tenosynovectomy (K028)	0.683
Tenolysis (K035)	0.617
Tendon transfer (K040)	0.582
Capsular release of elbow, wrist, and hand (K076-2)	0.462
Arthroplasty of hand and digits (K080)	0.505
Rotator cuff repair (K080-3, 4)	0.616
Stabilization of shoulder (K080-5)	0.548
Osteotomy of wrist and forearm fracture malunion (K054, K057)	0.330
Arthroscopic synovectomy of wrist and hand (K066-2)	0.598
Carpal tunnel release (K093)	0.574
Ulnar nerve transposition for cubital tunnel syndrome (K197)	0.707
Fasciectomy of cord for Dupuytren contracture (K099-2)	0.576
Reconstruction of syndactyly or polydactyly (K100, 101)	0.456
Urgent or trauma	
Tendon suture (K037)	1.214
Stabilization of subluxated extensor tendons (K040-2)	0.842
Percutaneous pinning for fracture of digits (K045-3)	0.669
ORIF for forearm and wrist fractures (K046-2, K073-2)	0.789
ORIF for humerus and elbow fractures (K046-1, K073-1)	0.776
ORIF for scaphoid fractures (K046-2)	0.651
ORIF for fractures of digit (K046-3)	0.747
Finger amputation (K086, K087)	2.158
Replantation of finger (K088)	1.697
Nerve repair (K182)	1.361

In the first column, numbers starting with “K” are surgical billing codes used by the Japanese insurance system. ORIF, open reduction and internal fixation.

**Table 4:** Trends in the number of surgeries.

Surgical Procedure	Pre-Pandemic	Initial Phase	Later Phase
Elective			
Tenosynovectomy (K028)	6848.3	6902.7 (0.068)	7010.2 (0.917)
Tenolysis (K035)	146.7	143.5 (0.730)	143.1 (0.192)
Tendon transfer (K040)	347.7	341.6 (0.284)	338.0 (0.635)
Capsular release of elbow, wrist, and hand (K076-2)	173.6	175.7 (0.277)	173.7 (0.119)
Arthroplasty of hand and digits (K080)	464.8	424.6 (>1)	436.0 (>1)
Rotator cuff repair (K080-3, 4)	1748.8	1686.6 (0.603)	1711.8 (0.301)
Stabilization of shoulder (K080-5)	438.0	431.1 (0.333)	451.6 (0.597)
Osteotomy of wrist and forearm fracture malunion (K054, K057)	375.3	293.7 * (>1)	301.9 * (>1)
Arthroscopic synovectomy of wrist and hand (K066-2)	151.6	163.1 (0.664)	167.6 (>1)
Carpal tunnel release (K093)	3202.4	3130.0 (0.683)	3162.9 (0.228)
Ulnar nerve transposition for cubital tunnel syndrome (K197)	479.7	460.4 (>1)	450.6 (>1)
Fasciectomy of the cord for Dupuytren contracture (K099-2)	111.0	180.2 (>1)	192.0 (>1)
Reconstruction of syndactyly or polydactyly (K100, K101)	235.7	219.9 (0.999)	211.6 (>1)
Urgent or trauma			
Tendon suture (K037)	546.9	543.1 (0.473)	526.3 (>1)
Stabilization of subluxated extensor tendons (K040-2)	64.6	58.1 (>1)	59.3 (>1)
Percutaneous pinning for fracture of the digits (K045-3)	1314.8	1139.7 (>1)	1155.8 (>1)
ORIF for forearm and wrist fractures (K046-2, K073-2)	6037.0	6047.3 (0.771)	6108.6 (0.481)
ORIF for humerus and elbow fractures (K046-1, K073-1)	3000.1	2988.2 (0.460)	3017.0 (0.167)
ORIF for scaphoid fractures (K046-2)	185.1	170.6 (>1)	171.5 (0.798)
ORIF for fractures of a digit (K046-3)	715.4	634.5 (>1)	623.1 (>1)
Finger amputation (K086, K087)	394.3	856.7 * (>1)	880.1 * (>1)
Replantation of finger (K088)	35.7	37.8 (0.532)	37.7 (0.854)
Nerve repair (K182)	188.4	180.1 (>1)	173.8 (>1)

In the first column, numbers starting with “K” are surgical billing codes used by the Japanese insurance system. In the last two columns, values in parentheses represent effect sizes relative to the pre-pandemic period obtained with a post-hoc test. ORIF, open reduction and internal fixation. * Indicates a significant difference compared to pre-pandemic level.

**Table 5:** Ratio of outpatient surgeries.

Surgical Procedure	Pre-Pandemic	Initial Phase	Later Phase
Elective			
Tenosynovectomy (K028)	90.6%	90.9% (0.730)	91.0% (>1)
Tenolysis (K035)	31.2%	33.4% (0.740)	33.5% (>1)
Tendon transfer (K040)	8.9%	11.1% * (>1)	11.7% * (>1)
Capsular release of elbow, wrist, and hand (K076-2)	14.9%	15.9% (0.543)	16.7% (>1)
Arthroplasty of hand and digits (K080)	7.8%	9.9% * (>1)	9.7% * (>1)
Rotator cuff repair (K080-3, 4)	0.0%	0.0%	0.0%
Stabilization of shoulder (K080-5)	0.0%	0.0%	0.0%
Osteotomy of wrist and forearm fracture malunion (K054, K057)	0.0%	0.0%	0.0%
Arthroscopic synovectomy of wrist and hand (K066-2)	0.0%	0.0%	0.0%
Carpal tunnel release (K093)	63.0%	65.1% * (>1)	65.8% * (>1)
Ulnar nerve transposition for cubital tunnel syndrome (K197)	11.0%	14.2% * (>1)	14.1% * (>1)
Fasciectomy of the cord for Dupuytren contracture (K099-2)	20.3%	25.2% (>1)	25.7% * (>1)
Reconstruction of syndactyly or polydactyly (K100, K101)	0.0%	0.0%	0.0%
Urgent or trauma			
Tendon suture (K037)	30.0%	30.6% (0.761)	30.4% (>1)
Stabilization of subluxated extensor tendons (K040-2)	46.6%	52.1% (>1)	53.9% * (>1)
Percutaneous pinning for fracture of the digits (K045-3)	63.5%	61.5% (>1)	61.8% (>1)
ORIF for forearm and wrist fractures (K046-2, K073-2)	4.3%	5.1% * (>1)	5.5% * (>1)
ORIF for humerus and elbow fractures (K046-1, K073-1)	0.4%	0.5% (0.162)	0.5% (0.616)
ORIF for scaphoid fractures (K046-2)	22.0%	23.8% (>1)	24.4% (>1)
ORIF for fractures of a digit (K046-3)	30.3%	31.3% (0.700)	31.6% (>1)
Finger amputation (K086, K087)	36.5%	19.9% * (>1)	19.2% * (>1)
Replantation of finger (K088)	0.0%	0.0%	0.0%
Nerve repair (K182)	22.8%	24.3% (0.788)	23.4% (0.672)

In the first column, numbers starting with “K” are surgical billing codes used by the Japanese insurance system. In the last two columns, values in parentheses represent effect sizes relative to the pre-pandemic period obtained with a post-hoc test. ORIF, open reduction and internal fixation. * Indicates a significant difference compared to pre-pandemic level.

## Data Availability

All the data used in this study are available on the website of the Ministry of Health, Labour, and Welfare of Japan.
